# Benefits of chemical sugar modifications introduced by click chemistry for glycoproteomic analyses

**DOI:** 10.1021/jasms.1c00084

**Published:** 2021-04-19

**Authors:** Beatriz Calle, Ganka Bineva-Todd, Andrea Marchesi, Helen Flynn, Mattia Ghirardello, Omur Y. Tastan, Chloe Roustan, Junwon Choi, M. Carmen Galan, Benjamin Schumann, Stacy A. Malaker

**Affiliations:** 1 Chemical Glycobiology Laboratory, The Francis Crick Institute, 1 Midland Road, NW1 1AT, London, United Kingdom; 2 Department of Chemistry, Imperial College London, 80 Wood Lane, W12 0BZ, London, United Kingdom; 3 Proteomics Science Technology Platform, The Francis Crick Institute, NW1 1AT London, United Kingdom; 4 School of Chemistry, Cantock’s Close, University of Bristol, BS8 1TS, United Kingdom; 5 Structural Biology Science Technology Platform, The Francis Crick Institute, NW1 1AT London, United Kingdom; 6 Department of Molecular Science and Technology, Ajou University, Suwon 16499, Republic of Korea; 7 Department of Chemistry, Yale University, 275 Prospect Street, New Haven, CT 06511, United States

## Abstract

Mucin-type O-glycosylation is among the most complex post-translational modifications. Despite mediating many physiological processes, O-glycosylation remains understudied compared to other modifications, simply because the right analytical tools are lacking. In particular, analysis of intact O-glycopeptides by mass spectrometry is challenging for several reasons; O-glycosylation lacks a consensus motif, glycopeptides have low charge density which impairs ETD fragmentation, and the glycan structures modifying the peptides are unpredictable. Recently, we introduced chemically modified monosaccharide analogs that allowed selective tracking and characterization of mucin-type O-glycans after bioorthogonal derivatization with biotin-based enrichment handles. In doing so, we realized that the chemical modifications used in these studies have additional benefits that allow for improved analysis by tandem mass spectrometry. In this work, we built on this discovery by generating a series of new GalNAc analog glycopeptides. We characterized the mass spectrometric signatures of these modified glycopeptides and their signature residues left by bioorthogonal enrichment reagents. Our data indicate that chemical methods for glycopeptide profiling offer opportunities to optimize attributes such as increased charge state, higher charge density, and predictable fragmentation behavior.

## Introduction

Protein glycosylation is one of the most complex post-translational modifications (PTMs) and is attached to proteins via Asn (N-linked) or Ser/Thr (O-linked) side chains.^1^ N-glycosylation is broadly grouped into three main categories: complex, hybrid, and high-mannose, and is found on predictable consensus peptide sequons (N_X_S/T).^2^ Though, while the presence of this sequon increases efficiency of glycosylation at the Asn, it does not necessarily guarantee that the site will be modified. Further, some non-canonical N-linked sequons have been identified, such as N_X_C, which adds complexity to N-glycoproteomic analyses.^3,4^ That said, N-glycopeptides can be sequenced by traditional collision-based methods, and search algorithms can often assign the sequence and associated glycan.^5,6^ Because of this, N-glycosylation is more straightforward to study by mass spectrometry. O-glycosylation, however, defies all of these conveniences. O-glycans can reside on any Ser or Thr residue, complicating site prediction. The most common type, extracellular mucin-like O-glycosylation, features an initiating α-D-GalNAc-O-Ser/Thr (GalNAc = D-*N*-acetylgalactosamine) linkage that can be extended in up to eight different core structures.^7,8^ Thus, O-glycan structures are extremely variable, leading to a large number of possible glycan and peptide combinations. Compounding this issue is that several S/T residues can be found in each O-glycopeptide, making site-localization more difficult. The variability in glycan structure and number of possible O-glycosites often leads to inaccurate assignment of O-glycopeptides by search algorithms.^9^ Overall, O-glycosylation is one of the most challenging PTMs to study by mass spectrometry.^10,11^

That said, the field of O-glycoproteomics has seen an explosion of technical advances in the last decade.^8,11–16^ Generally, glycoproteins are enriched using lectins, antibodies, and/or solid-phase extraction (i.e. hydrophilic interaction chromatography).^17^ After digestion, O-glycopeptides are separated via reverse-phase HPLC and introduced to the mass spectrometer, where they are often subjected to higher energy collision dissociation (HCD).^18^ Here, the bombardment of the glycopeptide with nitrogen atoms invariably generates a “HexNAc fingerprint” that consists of the HexNAc (GalNAc or GlcNAc = D-*N*-acetylglucosamine) oxonium ion along with five fragments from this ion.^19,20^ Commonly, the HexNAc fingerprint is employed to trigger product-dependent (pd) electron transfer dissociation (ETD) or ETD with supplemental activation (EThcD), which is then used to site-localize the modification along the peptide backbone.^21^ Together, the information gathered in HCD and ET(hd)D spectra make it possible to sequence the peptide, identify the glycan composition, and site-localize the modification(s).^4,22^

**Figure 1:**
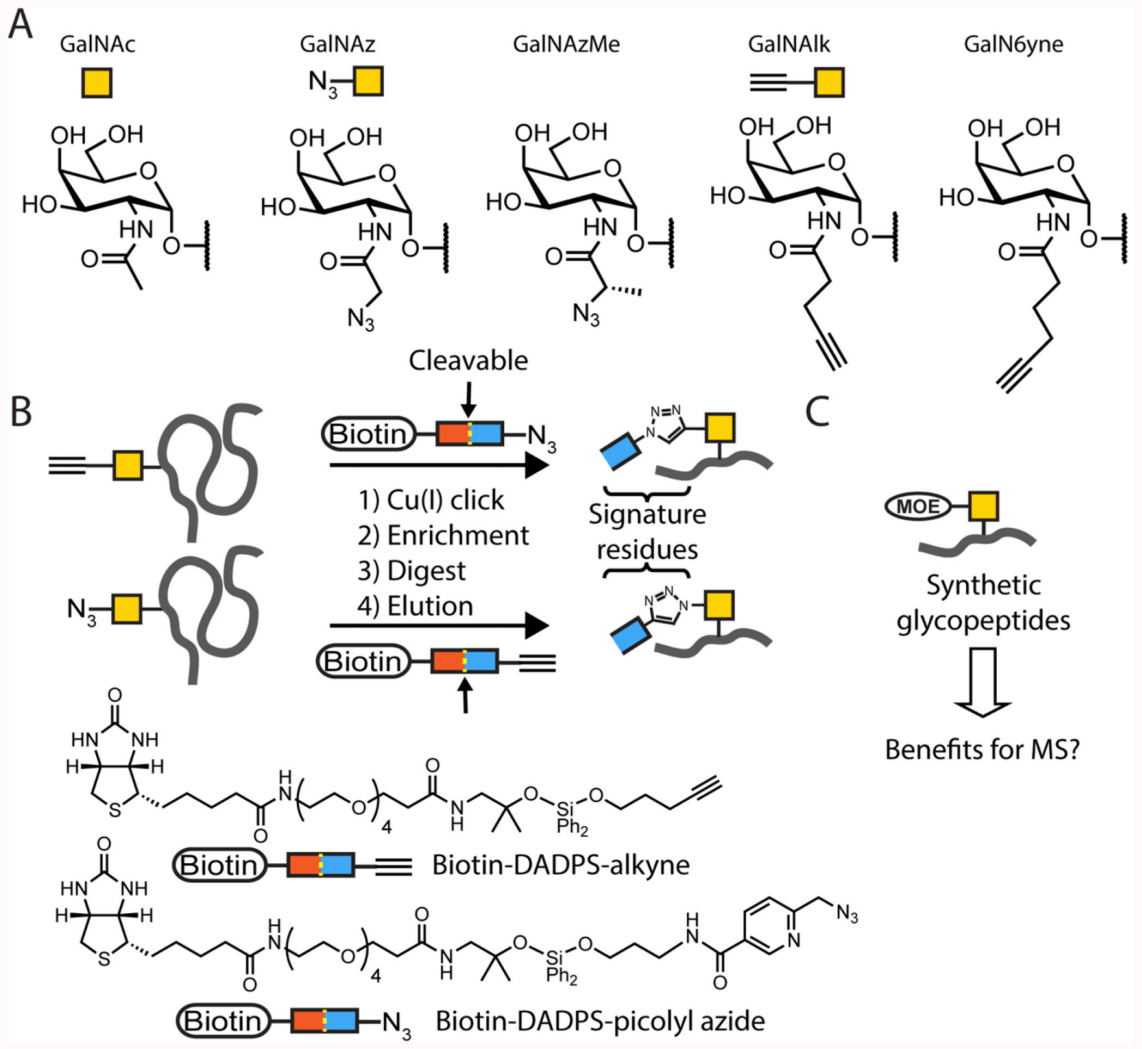
Overview of experimental rationale. (A) Overview of bioorthogonal GalNAc analogs referred to in this work. The sugars GalNAc, GalNAz, and GalNAlk used in synthetic glycopeptides here are shown with their assigned symbol. (B) Typical workflow of MOE-glycoproteomics, here exemplified for glycoproteins containing azide (e.g. GalNAz, GalNAzMe) or alkyne (e.g. GalNAlk, GalN6yne) functionalities. In previous experiments, we reacted glycopeptides with the depicted cleavable biotin handles Biotin-DADPS-alkyne and Biotin-DADPS-picolyl azide. This left a MOE signature residues on the glycopeptides that we used to aid in MS analysis. (C) Experimental rationale herein. We generated synthetic glycopeptides with defined MOE signature residues in order to observe their behavior in MS. DADPS = dialkoxydiphenylsilane.

Metabolic oligosaccharide engineering (MOE), a recent addition to the field of intact O-glycoproteomics, is emerging as an important strategy to profile mucin-type O-glycans.^23,24^ In the MOE strategy, monosaccharides are chemically modified with tags that are stable towards the environment in the living cell, but reactive towards methods of bioorthogonal (or “click”) chemistry such as copper-mediated azide-alkyne cycloaddition (CuAAC).^25^ A collection of bioorthogonal GalNAc-based monosaccharides relevant to this work is shown in Figure 1A. Tagged monosaccharides are rendered cell-permeable by derivatizing polar groups such as hydrophobic esters and thioesters. After cell entry and removal of esters by cytosolic esterases, tagged monosaccharides are metabolically activated into nucleotide-sugars that can be used by glycosyltransferases.^23,24,26,49^ This process incorporates chemical tags into the glycoproteome of a living cell, allowing for the introduction of enrichment reagents such as biotin by CuAAC.^27^ The ensuing enrichment, tryptic digest, and glycoproteomic analysis have enabled the assignment of glycosylation sites and glycan structures, but are still hampered by ion suppression in the presence of non-glycosylated peptides.^27^ Following the initial implementation of MOE reagents, another improvement came through the use of specialized cleavable biotin enrichment reagents. These handles allow for selective elution of O-glycopeptides from the enrichment matrix (typically neutravidin) after non-glycosylated peptides have been removed by an on-bead protease digest (Figure 1B).^27–29^ While this type of selective glycopeptide enrichment has led to unique insight into the glycoproteome, some of the fundamental challenges such as glycan heterogeneity and sub-stoichiometric abundance call for further innovation in the field of chemical glycoproteomics.^30^

We recently developed chemically tagged GalNAc derivatives that were selectively introduced into mucin-type O-glycans.^27–29^ Using MOE in conjunction with acid-cleavable, bioorthogonal-biotin reagents allowed profiling of mucin-type O-glycosylation by intact glycoproteomic analysis. Instead of the typical HexNAc fingerprint, the covalent attachment of enrichment handles left predictable MOE signature residues and reliable oxonium ion fingerprints (Figure 1B). The new oxonium ions facilitated automated ETD triggering, which then allowed us to manually assign sites of glycosylation. In addition to the oxonium ions, we serendipitously observed that the MOE signature residues displayed unique mass spectrometric signatures with additional benefits in increased peptide charge and the presence of ETD fingerprint ions.^27–29^

Here, we describe and quantify the benefits of chemical signature residues in MOE as a basis to systematically compare a panel of new bioorthogonal reagents introduced into synthetic glycopeptides (Figure 1C). While all glycopeptide derivatives generated oxonium ions that were able to trigger ETD, we found that the introduction of increasingly basic or charged functionalities in MOE signature residues led to higher charge states in MS1 spectra. Deliberate incorporation of a stable positive charge through imidazolium tag (ITag)-containing reagents resulted in an additional 70-99% increase in relative abundance of the highest charge state.^31–33^ Further, the elevated charge density increased the dissociation efficiency by ETD, leading to high peptide sequence coverage. Ultimately, we expect these reagents will prove extremely useful in complex O-glycoproteomics experiments.

## Methods

For information regarding synthesis of molecules and chemoenzymatic synthesis of glycopeptides, please see the Supplementary Information.

Biotin-DADPS-picolyl-azide (**SI-6**) was purchased from Sussex Research (Ottawa, Canada). Biotin-DADPS-alkyne (**SI-3**) was purchased from Click Chemistry Tools (Scottsdale, USA).

### Purification of glycopeptides by solid phase extraction

Following chemoenzymatic synthesis on a 1 nmol scale, glycopeptides were dried by Speedvac and resuspended in 1 mL 0.1% (v/v) aqueous formic acid. A Strata-X (60 mg /mL) Phenomenex solid phase extraction column was used for purification of the synthetic glycopeptides. The column was primed by washing with 1 mL acetonitrile and 1 mL 0.1% (v/v) aqueous formic acid. The samples were then loaded onto the column. The column was washed with 1 mL 0.1% (v/v) aqueous formic acid and the synthetic glycopeptides eluted from the column with 300 μL 80% acetonitrile/ 20% water (v/v) with 0.1% formic acid. The samples were then aliquoted into 100 pmol aliquots and dried by Speedvac.

### Mass spectrometry of glycopeptides

Samples were analysed by online nanoflow LC-MS/MS using an Orbitrap Fusion Lumos mass spectrometer (Thermo Scientific) coupled to an Ultimate 3000 RSLCnano (Thermo Scientific). Sample (1-10 μL, approx. 500 fmol glycopeptide per run in 0.1% aqueous formic acid, FA) was loaded via autosampler into a 20 μL sample loop and pre-concentrated onto an Acclaim PepMap-100 75 μm x 2 cm nanoviper trap column with loading buffer, 2% v/v acetonitrile, 0.05% v/v trifluoroacetic acid, 97.95% water (Optima grade, Fisher Scientific) at a flow rate of 7 μL/min for 6 min in the column oven held at 40 °C. Peptides were gradient eluted onto a PepMap RSLC C18 75 μm x 50 cm, 2 μm particle size, 100Å pore size, reversed phase EASY-Spray analytical column (Thermo Scientific) at a flow rate of 275 nL/min and with the column temperature held at 40 °C, and a spray voltage of 2400 V using the EASY-Spray Source (Thermo Scientific). Gradient elution buffers were A 0.1% v/v FA, 5% v/v DMSO, 94.9% v/v water and B 0.1% v/v FA, 5% v/v DMSO, 20% v/v water, 74.9% v/v acetonitrile (all Optima grade, Fisher Scientific aside from DMSO, Honeywell Research Chemicals). The gradient elution profile was 2% B to 40% B over 27 minutes. The instrument method used an MS1 Orbitrap scan resolution of 120,000 at FWHM m/z 200, quadrupole isolation, mass range 300-1500 m/z, RF Lens 30%, Standard AGC target, maximum injection time 50 ms and spectra were acquired in profile. Monoisotopic Peak Determination was set to the peptide mode, and only precursors with charge states 2-6 were permitted for selection for fragmentation. Dynamic Exclusion was enabled to exclude after n=3 times within 10 s for 10 s with high and low ppm mass tolerances of 10 ppm. HCD was performed on all selected precursor masses using a cycle time-based data dependant mode of acquisition set to 3 s. MS2 scans were acquired in the Orbitrap at a resolution of 30000 FWHM m/z 200, following HCD fragmentation with fixed collision energy of 28% after quadrupole isolation with an isolation window width of 2 m/z. The parameters used for the HCD MS2 scan were auto scan range mode, Standard AGC target, custom maximum injection time 54 ms and the scan data was acquired in centroid mode. Each experiment was performed twice; the first used an HCD-pd-ETD instrument method, whereby ETD fragmentation was only performed if precursors were within the precursor selection range m/z 300-1000 and if one of the following list of mass trigger ions were present in the HCD MS2 spectra +/-0.1 m/z and above the relative intensity threshold of 10% (126.055, 138.0549, 144.0655, 168.0654, 186.076, 204.0855, 245.0886, 227.0780, 329.1461, 311.1355, 348.1308, 330.1202, 380.1808, 362.1702, 242.1028, 224.0923, 343.1618, 325.1512, 477.2098, 459.1992, 408.2121, 390.2016 m/z or top speed HCD/ETD toggle). The second run used an HCD/ETD toggle at top speed for 3 s. ETD MS2 scans were recorded in the ion trap with rapid scan rate following quadrupole isolation with an isolation window width of 3 m/z. ETD activation used calibrated charge-dependent ETD parameters, the automatic scan range mode was used and parameters were set for the Standard AGC target, maximum injection time 100 ms and scan data acquired in centroid mode. Note: ETD for ITag glycopeptides triggered on ^13^C satellite peaks – the correct trigger ions to use are 379.1730 m/z (GalNAc-**4**) and 407.2043 m/z (GalNAc-**7**). High resolution ETD experiments were performed on an Orbitrap Tribrid Eclipse, where the ETD parameters were as follows: Orbitrap detection at 7500 resolution, 100% AGC, calibrated reaction times, and 100 ms injection time. All glycopeptides were manually analyzed using Thermo Xcalibur software.

## Results and Discussion

### Analysis of Chemically Modified Glycopeptides Enriched from Complex Cell Lysates

**Figure 2:**
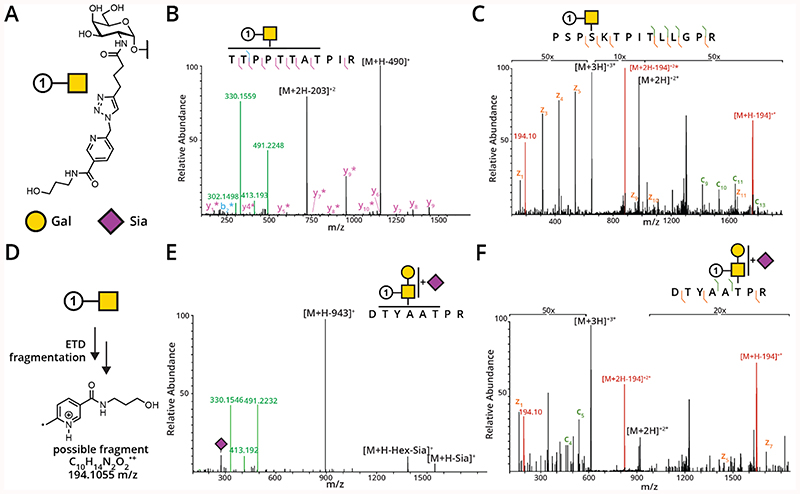
Unique mass spectrometric behavior of GalN6yne-modified glycopeptides from previous chemical glycoproteomics experiments. (A) MOE signature residues **1** is produced in an MOE-glycoproteomics experiment incorporating GalN6yne as the sugar. (B) The GalNAc-**1** (mass: 491.2249) oxonium ion is detected at 491.2248 m/z. Another abundant ion is detected at 330.1559 m/z. (D) Example ETD spectra demonstrating the presence of the fingerprint ion at 194.10 m/z; the glycopeptide originated from van Willebrand factor C. (D) Proposed structure of the 194.10 ion. (E) HCD and (F) ETD spectra demonstrating that GalN6yne is extended by naturally occurring monosaccharides in the living cell; this GalN6yne-Hex-NeuAc glycopeptide originates from glycophorin A. Legend: b ions are indicated in blue, y ions in pink, z ions in orange, and c ions in dark green. Oxonium ions are indicated in light green, and ETD fingerprint ions are denoted in red. Gal (yellow circle) = D-Galactose; Sia (purple diamond) = D-*N*-acetylneuraminic acid.

While analyzing tandem mass spectrometry data from our recent MOE experiments using chemically tagged analogs of GalNAc,^28,29^ we observed unique mass spectrometric signatures that we harnessed to confidently assign glycopeptide identities. For instance, MOE signature residues **1** derived from the alkyne-containing sugar GalN6yne reacted with the commercial, acid-cleavable enrichment handle Biotin-DADPS-picolyl azide (Figure 2A) led to unique fingerprint ions after fragmentation by HCD. As demonstrated in Figure 2B, the peptide TTPPTTATPIR from the secreted protein fibronectin was modified by GalNAc-**1**; the ensuing oxonium ion was detected as the modification at 491.2248, as well as an abundant fragment ion at 330.1559. Some less abundant ions were detected at 413.193 (loss of C_2_H_6_O_3_) and 302.1498 (loss of water). We hypothesize that these additional ions stem from fragmentation of the picolyl moiety in GalNAc-**1**, demonstrating the potential of MOE signature residues on validating HCD spectra.

In related studies using the azide-containing sugars GalNAz and GalNAzMe and the enrichment handle Biotin-DADPS-alkyne (Figure 2A), we also observed unique oxonium ion patterns (Figure S1, Supporting Information).^29^ The oxonium ion for GalNAc-**2** (from GalNAz) was detected primarily at 329.1455, along with two ions corresponding to the loss of one (311.1348) or two (293.1243) water molecules (Figure S1A, Supporting Information). Finally, an oxonium ion corresponding to GalNAc-**2Me** (from GalNAzMe) was detected at 343.1602 (Figure S1B, Supporting Information). Similar to GalNAc-**2**, two water losses were detected, at 325.1494 and 307.1389. A second abundant ion was detected at 178.1972. These ions were reliably present on many of the modified peptides throughout our analyses and, as such, could be used as product-dependent (pd) ETD triggers instead of the canonical “HexNAc fingerprint” ions.

With this information in-hand, we repeated the above analyses using an HCD-pd-ETD instrument method.^28,29^ In the case of GalNAc-**2** and GalNAc-**2Me** peptides, the ETD spectra were not unlike unmodified GalNAc glycopeptides, in that the MOE signature residues remained intact during fragmentation. While analyzing the GalNAc-**1** samples (which have larger MOE signature residues), we noticed that a peak at 194.10 m/z was present in all of the ETD spectra (Figure 2C). We propose that this ion is the result of electron transfer to the MOE signature residues, which triggers fragmentation (Figure 2D). In addition to the ion at 194.10 m/z, a small molecule fragmentation pattern is present from the +2 masses; this is observed as a “loss” of 97 from the [M+2H]^+2^ ion. These ions were reliably present in all of the GalNAc-**1** containing glycopeptide ETD spectra (Figure 2C, D, and Supporting Information, Figure S2). We are currently working with glycoproteomic platforms so as to incorporate these ions into the scoring algorithms, due to their consistent presence in ETD spectra. For example, in a representative dataset containing 49 GalNAc-**1** modified glycopeptides, the 194 signature residue was present in every ETD spectrum when the precursor charge was ≥3 (42/42, 100%) and was weakly detected in most ETD spectra when the precursor charge was 2 (5/7, 71%) Thus, we envision this will lead to more confident site-assignment of glycopeptides.

A seminal strategy in the field of glycoproteomics was developed by Clausen and colleagues to facilitate O-glycan analysis by simplifying their biosynthesis. This was achieved through knock-out of a chaperone that is needed for an extension step of the initial GalNAc moiety.^34,35^ While these “SimpleCells” were an important contribution to our understanding of what sites are modified by O-glycosylation, the information beyond GalNAc was lost by nature of the method. In MOE experiments, we and others have observed that chemically modified GalNAc analogs can be elaborated by downstream glycosylation.^27–29,36,37^ As demonstrated in Figure 2E and Supporting Information Figure S3A, B, extension of the GalNAc analogs GalN6yne, GalNAz, and GalNAzMe could be observed in our MOE systems by virtue of defined HCD fragment ions. It is currently not known whether downstream glycosylation of these analogs proceeds with the same fidelity as for native GalNAc. However, recent findings suggest that monosaccharide analogs are incorporated into various glycan sub-types without a notable chain-terminating effect.^28,29,36,37^

### Generation of new chemoenzymatically synthesized glycopeptides for MS analysis

**Figure 3:**
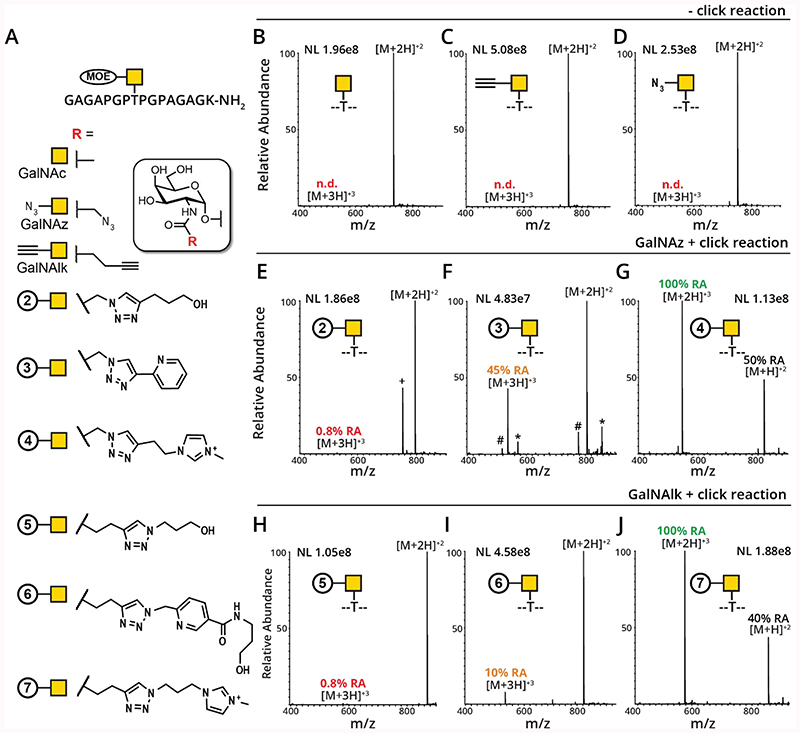
Modified sugar substrates may impart charge to the glycopeptide. (A) Glycopeptides prepared by chemoenzymatic synthesis. CuAAC was used to introduce MOE signature residues **2**-**7**. (see Supporting Information for details). Glycopeptides were analyzed on an Orbitrap Fusion Lumos. (B-D) Analysis of unclicked substrate peptides containing GalNAc, GalNAz, and GalNAlk. Glycopeptides were detected as [M+2H]^+2^ species; the [M+3H]^+3^ ions were not detected (n.d.). (E-G) Analysis of glycopeptides containing MOE signature residues **2**-**4**. (E) GalNAc-**2** was detected as a [M+2H]^+2^ species; the [M+3H]^+3^ ion was present at 0.8% relative abundance (RA). The + indicates unreacted GalNAz-glycopeptide, which did not fully separate chromatographically from the modified species. (F) GalNAc-**3** was detected as a [M+2H]^+2^ species; the [M+3H]^+3^ ion was present at 45% RA.. The # and * indicate the loss of Gly and addition of Thr, respectively, likely an effect of peptide synthesis as opposed to side reactions. (G) GalNAc-**4** was detected as a [M+2H]^+3^ species; the [M+H]^+2^ ion was present at 50% RA (H-J) Analysis of of glycopeptides containing MOE signature residues **5**-**7**. (E) GalNAc-**5** was detected as a [M+2H]^+2^ species; the [M+3H]^+3^ ion was present at 0.8% RA. (F) GalNAc-**6** was detected as a [M+2H]^+2^ species; the [M+3H]^+3^ ion was present at 10% RA. (G) GalNAc-**7** was detected as a [M+2H]^+3^ species; the [M+H]^+2^ ion was present at 40% RA.

Encouraged by the beneficial effect imparted by bioorthogonal sugars in MS analysis, we sought to undertake a comparative study of defined MOE signature residues in synthetic glycopeptides. The ideal MOE signature residues would combine the attributes outlined in Figure 2 (HCD oxonium ions, ETD fingerprint ions, and ability to be detected even after extension by natural glycosyltransferases) but would also incorporate an increase in charge state/density that should lead to augmented dissociation efficiency and fragment ion detection using ETD fragmentation. With this in mind, we designed a panel of glycopeptides outlined in Figure 3A (Supporting Information, Schemes S1–S3). The peptide GAGAPGPTPGPAGAGK-NH_2_ carries a single Thr that can be modified with a GalNAc or a suitable bioorthogonal analog by the human glycosyltransferase GalNAc-T2. The engineered enzyme will transfer either GalNAc, GalNAz, or GalNAlk using the corresponding UDP-sugars as substrates.^26,28,29,38^ To evaluate the effect on MOE signature residues on performance in mass spectrometry, we derivatized the azide and alkyne moieties in GalNAz and GalNAlk, respectively, using CuAAC. We chose to generate MOE signature residues with increasingly basic functionalities: while all contained a triazole as a remnant from the CuAAC reaction, we chose to compare simple aliphatic alcohols (GalNAc-**2** and GalNAc-**5**) with additional pyridine or picolyl moieties (GalNAc-**3** and GalNAc-**6**, respectively). We further chose an imidazolium-containing residue with a stable positive charge (GalNAc-**4** and GalNAc-**7**), as such reagents (ITags) have been used with great success to analyze and purify glycans from synthetic or cell-derived sources.^39–41^ We took inspiration from strategies to improve ionization in isolated natural compounds, peptides, and glycopeptides, among others.^42–45,48^

The glycopeptides were analyzed using an Orbitrap Fusion Lumos Tribrid mass spectrometer (Thermo Fisher) followed by manual analysis with Xcalibur software. In looking at the MS1 intensities, we confirmed that MOE signature residues introduce positive charge to the glycopeptides: with higher basicity of the chemical handle comes an increase in charge state. For instance, glycopeptides containing GalNAc (Figure 3B) or unclicked GalNAlk (Figure 3C) and GalNAz (Figure 3D) were all detected solely as a +2 ion; the +3 ion was not detected (n.d.) for any of these glycopeptides. The GalNAz-modified peptide was then converted to the derivatives GalNAc-**2**, GalNAc-**3** or GalNAc-**4** (Figure 3E-G). GalNAc-**2** was primarily detected as a [M+2H]^+2^ species, but the [M+3H]^+3^ was weakly present at 0.8% relative abundance (RA, Figure 3E). This is likely because the triazole moiety is basic enough to accept an additional proton at low abundance. GalNAc-**3,** containing a pyridine substituent, with an additional basic nitrogen imparted a significant increase in the [M+3H]^+3^ abundance; here, the +3 ion increases to 45% RA (Figure 3F). Most transforming with respect to charge state, though, was the ITag glycopeptide (GalNAc-**4**), where the [M+2H]^+3^ ion dominated the MS1 abundance (100% RA) and the +2 ion was less abundant (50% RA). Similarly, the GalNAlk-modified peptide was reacted to give GalNAc-**5**, GalNAc-**6** and GalNAc-**7**. GalNAc-**5** induced a weak charge increase to the glycopeptide (Figure 3H). GalNAc-**6** induced a higher [M+3H]^+3^ abundance (10% RA, Figure 3I), highlighting the usefulness of the enrichment handle Biotin-DADPS-picolyl azide for mass spectrometry applications. The ITag (GalNAc-**7**) again increased the [M+2H]^+3^ ion to 100% RA (Figure 3J). Given that ETD relies on charge density,^21^ and that ETD is necessary for accurate site-assignment of O-glycosites,^5^ we envision that reacting MOE glycopeptides with Biotin-DADPS-picolyl azide (for enrichment) or ITag (without enrichment) bioorthogonal reagents will result in a higher number of beneficial ETD spectra. As a corollary, this should also result in better site localization of glycans along the peptide backbone, even in complex samples. Of note, the click reaction using pyridine-containing azides to generate compounds GalNAc-**3** and GalNAc-**6** is likely more efficient than for aliphatic azides due to the faster reaction kinetics.^46^

### MOE signature residues provide reliable HCD oxonium ions

**Figure 4:**
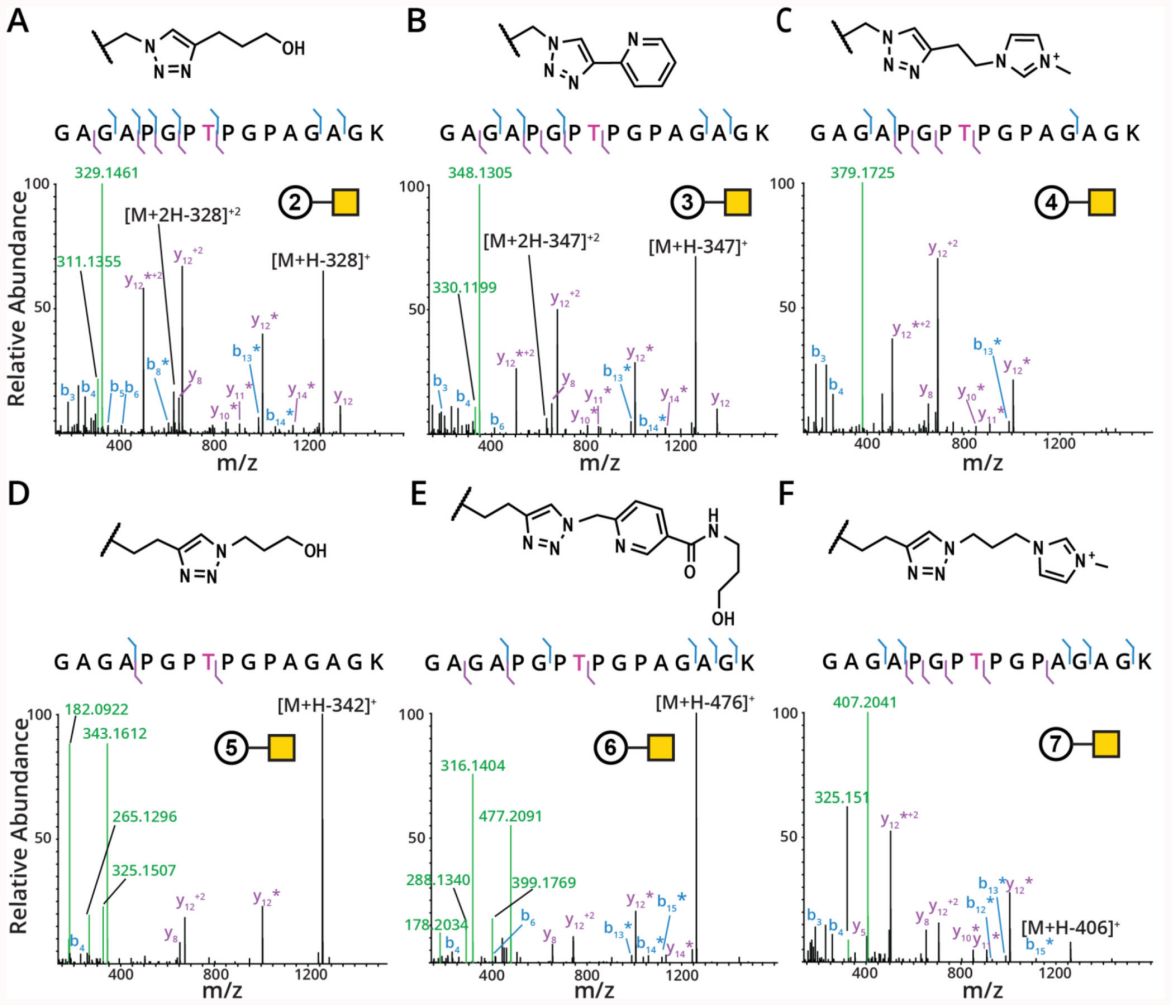
GalNAc analogs have altered oxonium ion patterns. Chemoenzymatically generated synthetic glycopeptides were reacted with reagents **2-7**, then subjected to HCD fragmentation on an Orbitrap Fusion Lumos. (A-C) GalNAz-glycopeptides were reacted with reagents **2-4** using CuAAC. (D-F) GalNAlk-glycopeptides were reacted with reagents **5-7**. Legend: b ions are indicated in blue, y ions in purple, and oxonium ions are indicated in light green. Asterisks indicate the fragment ion is detected without the glycan moiety attached. The pink “T” signifies the site of modification.

We next asked how the glycopeptides containing MOE signature residues fragmented by higher-energy collision HCD. Unsurprisingly, as in GalNAc-containing O-glycopeptides, one of the most abundant ions present is the peptide sans glycan, denoted as [M+H-glycan]^+^ (Figure 4). Additionally, we were able to detect a large number of fragment ions, most of which were dissociated from the modification, as denoted by an asterisk. As before, the main distinction between unmodified GalNAc and analogs containing MOE signature residues was that the oxonium ions were different from the traditional HexNAc fingerprint. For example, the HCD spectrum for GalNAc-**2** (Figure 4A) had a dominant oxonium ion at 329.1461 (M+H), with another, less abundant ion at 311.1355 (M+H-H_2_O). The oxonium ions for GalNAc-**3** were similar – one at 348.1305 (M+H) and another at 330.1199, corresponding to the loss of water (Figure 4B). GalNAc-**4**, though, had a single oxonium ion present at 379.1725 (M+H), Figure 4C). GalNAc-**5** had an abundant ion at 343.1612 (M+H) and an ion representing the water loss (325.1507), but also a very abundant ion at 182.0922 (loss of hexosamine) and a slightly less abundant ion present at 265.1296 (Figure 4D). Unfortunately, despite the fact that a similar number of ions were used to generate all MOE signature residue HCD spectra shown here, GalNAc-**5** has particularly low fragmentation efficiency. Thus, this GalNAc-**5** may not be ideal for glycoproteomic analyses. Similarly, GalNAc-**6** had an abundant ion at 477.2091 (M+H) along with an even more abundant ion at 316.1405 corresponding to the loss of hexosamine (Figure 4E). Two less abundant ions also appeared at 288.1342 and 399.1774. Finally, the ITag modified glycopeptide containing GalNAc-**7** in Figure 4F fragmented such that only the M+H and the -H_2_O ions were detected at 407.2041 and 325.1510, respectively. These ions were enough to trigger ETD fragmentation on the precursor ions, and we expect these masses will be sufficient to use in more complex samples. Given the flexibility of instrumentation methods available, coupled with the high-resolution detection in an Orbitrap, even with a smaller number of oxonium ions it is possible to trigger ETD specifically when glycopeptides are present. See Table 1 for more detailed information regarding instrument method design and implementation. Of note, the HCD oxonium ions we observed for both ITag-derivatized GalNAc derivatives GalNAc-**4** (379.1725 m/z) and GalNAc-**7** (407.2041 m/z) were singly charged and missing the mass of one hydrogen from the calculated exact masses of 380.1808 (GalNAc-**4**) and 408.2121 (GalNAc-**7**). We hypothesize that HCD fragmentation leads to the net loss of a proton in the ITag MOE signature residues, and a similar behavior has previously been observed for imidazolium ions.^47^

### GalNAz-ITag glycopeptide ETD spectra contain an ETD fingerprint ion

**Figure 5:**
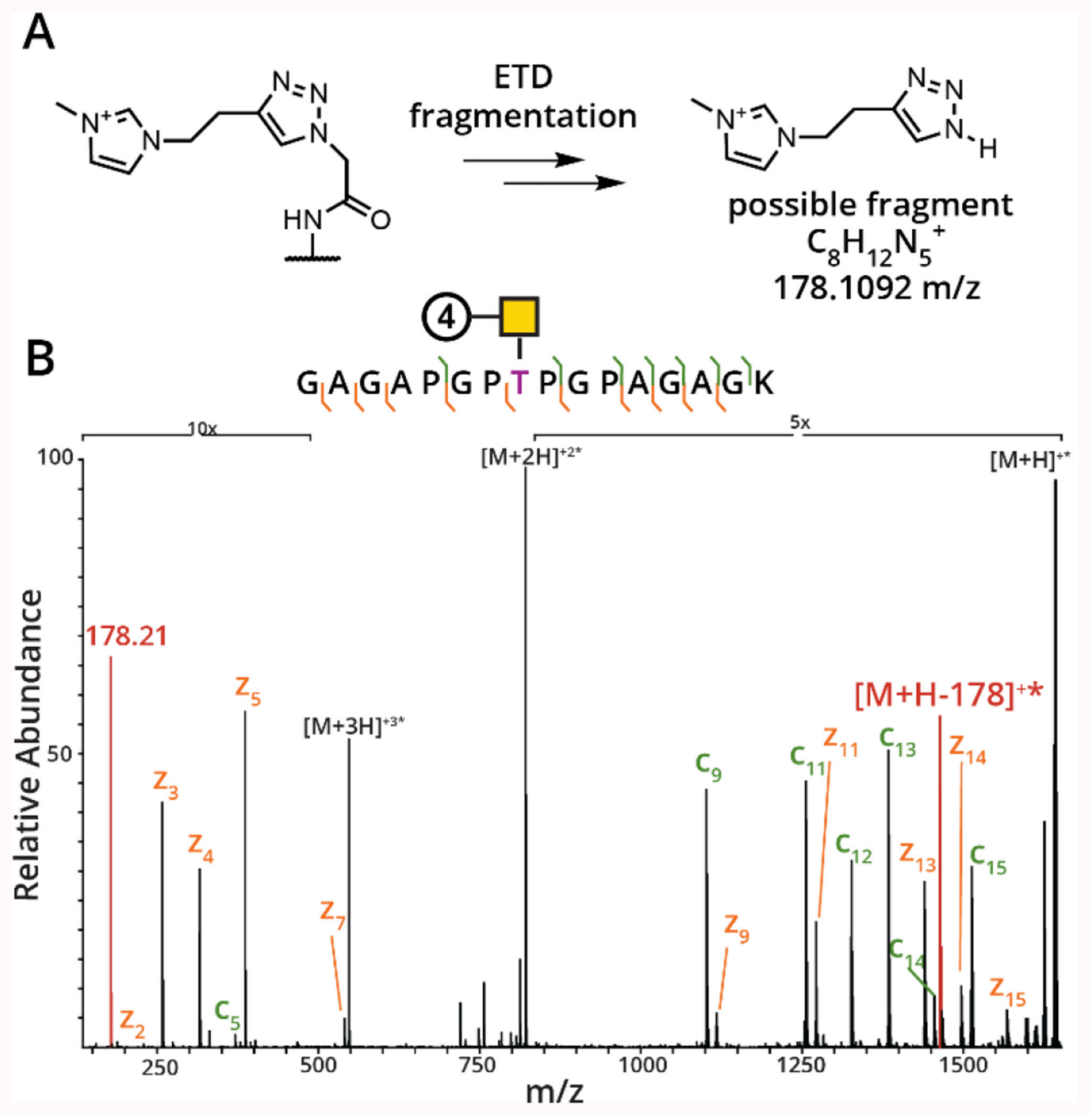
ETD spectrum of GalNAz-ITag glycopeptide. (A) Proposed fragmentation scheme for generation of the 178 and [M+H-178]^+^ ions. (B) Example [M+2H]+3 ETD spectrum of a Reagent **4** (ITag) modified glycopeptide. The site of modification is colored pink; z ions are denoted in orange, c ions in dark green, and ETD fingerprint ions in red.

Intrigued by the higher charge state imparted by the ITag reagents, we next investigated the ETD spectra of the glycopeptides containing GalNAc-**4** and GalNAc-**7**. As mentioned above, we were able to trigger ETD on all of the glycopeptides but were most curious about the behavior of stably charged ITag-glycopeptides. Perhaps unsurprisingly, the ITag-modified peptides fragmented well by ETD, as the high charge density imparted by the ITag increased fragmentation efficiency and our ability to detect fragment ions. Interestingly, GalNAc-**4** had an ETD fingerprint ion similar to the one found in Figure 2C-D. This fingerprint was present at both 178 m/z and was also observed as a small molecule fragmentation from the intact peptide. Our hypothesis for how this fragment is generated is depicted in Figure 5A. Here, electron transfer from the radical electron to the ITag reagent may cause dissociation of the triazole from the hexosamine but can be detected due to the positive charge present on the modification. A high-resolution ETD spectrum for accurate mass of the ETD fingerprint ion can be found in Supporting Information Figure 4. We found the accurate mass of the signature residue to be 178.1082, which is -5.6 ppm from the calculated fragment mass of 178.1092, thus supporting our proposed structure. An example low-resolution spectrum is shown in Figure 5B, where the ETD fragment and the small molecule fragmentation pattern are shown in red. Again, we envision that this unique fingerprint could be incorporated into current search algorithms as a confirmation that a glycopeptide is, in fact, present. Finally, as mentioned above, we observed excellent peptide sequence coverage, likely because of the increased charge density in these peptides. Ultimately, we believe the ITag reagents present a huge opportunity for intact O-glycoproteomics through increased charge states, a concomitant increase in ETD spectral quality, and ETD fingerprint ions. The mass spsectrometric details of checmial scars studied herein are shown in Table 1.

## Conclusions

Here, we demonstrate the benefits of using modified sugar analogs for glycoproteomic analysis, including increased peptide charge, unique oxonium ion fragmentations, ETD fingerprint ions, and the elongation of the GalNAc analog. We believe these unique features of our bioorthogonal reagents will find use in glycoproteomic analyses, especially if they can be incorporated into existing search algorithms. The field of chemical glycoproteomics is rapidly evolving, opening windows into understanding one of the most complex post-translational modifications and their associated enzyme families. This work represents a contribution toward that effort, demonstrating that unique sugar analogs have unique mass spectrometric properties that can be leveraged for higher charge, ETD triggers, and/or confident glycopeptide assignment.

## Supplementary Material

Figure S1, Supporting Information

## Figures and Tables

**Table 1:** Summary of mass spectrometric behaviour of glycopeptides containing MOE signature residues. For all glycopeptides except GalNAc-1, the underlying peptide was GAGAPGPT*PGPAGAGK-NH_2_, with T* indicating the glycosylation site. For GalNAc-1, the HCD and ETD ions were observed in our live cell experiments, thus, the MS1 RA is not directly comparable to the other glycopeptides.

Sugar	GalNAc derivative	MS1 ion RA	HCD fragment ions	ETD fingerprint ions
GalNAc	-	[M+2H]^2+^: 100% [M+3H]^3+^: n. d.	Canonical HexNAc fingerprint ions	n/a
GalNAz	GalNAz	[M+2H]^2+^: 100% [M+3H]^3+^: n. d.	245.0881, 227.0770, 167.0563	n/a
GalNAlk	GalNAlk	[M+2H]^2+^: 100% [M+3H]^3+^: n. d.	242.1025, 224.0919, 176.0707, 164.0706, 144.0656	n/a
GalNAc-**1**	GalN6yne-picolyl-triazole	n/a	491.2248, 330.1559, 413.1325, 302.1498	194.10
GalNAc-**2**	GalNAz-propanolyl-triazole	[M+2H]^2+^: 100% [M+3H]^3+^: 0.8%	329.1461, 311.1355	n/a
GalNAc-**3**	GalNAz-pyridyl-triazole	[M+2H]^2+^: 100% [M+3H]^3+^: 45%	348.1305, 330.1199	n/a
GalNAc-**4**	GalNAz-ITag-triazole	[M+H]^2+^: 50% [M+2H]^3+^: 100%	379.1725	178.11
GalNAc-**5**	GalNAlk-propanolyl-triazole	M+2H]^2+^: 100% [M+3H]^3+^: 0.8%	343.1612, 325.1507, 265.1296, 182.0922	n/a
GalNAc-**6**	GalNAlk-picolyl-triazole	[M+2H]^2+^: 100% [M+3H]^3+^: 10%	477.2091, 399.1769, 316.1404, 288.1340, 178.2034	n/a
GalNAc-**7**	GalNAlk-ITag-triazole	[M+H]^2+^: 40% [M+2H]^3+^: 100%	407.2041, 325.1510	n/a
