# Supplementary material for: Benefits of chemical sugar modifications introduced by click chemistry for glycoproteomic analyses
Source: J Am Soc Mass Spectrom. Author manuscript; Available in PMC 2021 Sep 3. (PMC7611619; doi:10.1021/jasms.1c00084)

**Supporting Information for:**

Benefits of chemical sugar modifications introduced by click chemistry for glycoproteomic analyses

Beatriz Calle^‡^, Ganka Bineva-Todd^‡^, Andrea Marchesi^‡^, Helen Flynn, Mattia Ghirardello, Omur Y. Tastan, Chloe Roustan, Junwon Choi, M. Carmen Galan, Benjamin Schumann^*^, Stacy A. Malaker^*^

^‡^ These authors contributed equally to the manuscript.

*Correspondence should be addressed to S.A.M. and B.S.

Email: stacy.malaker@yale.edu, b.schumann@imperial.ac.uk

**This PDF file includes:**

Supporting Information Figures S1 to S4

Scheme S1 to S3

Supplemental Methods

NMR spectra


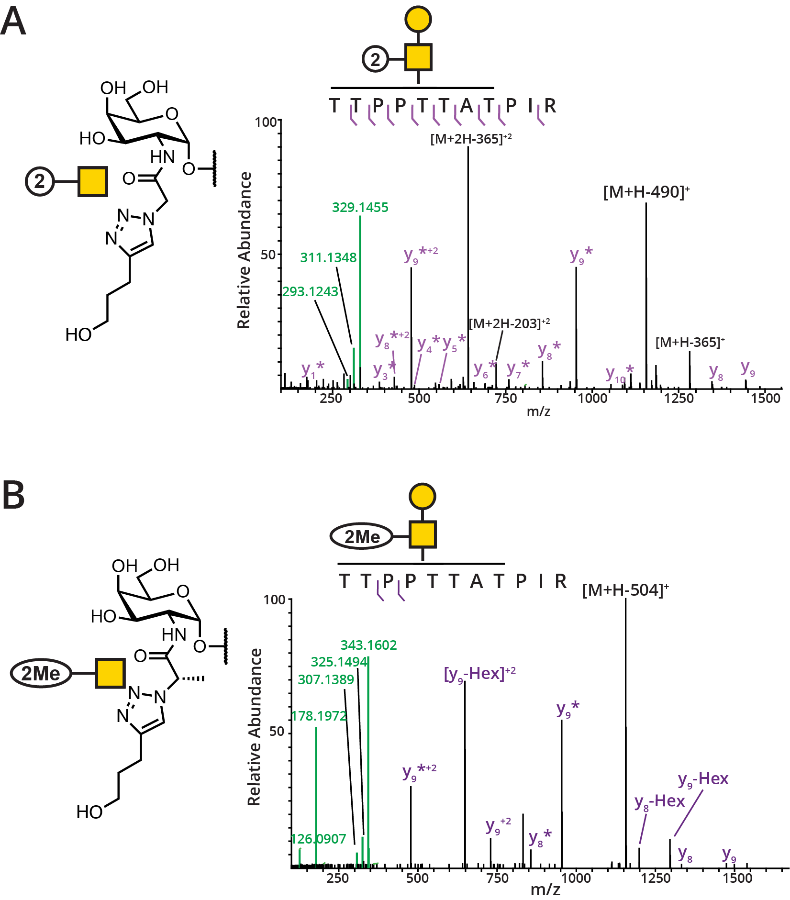


**Figure S1. Unique mass spectrometric behavior of GalNAz. And GalNAzMe-modified glycopeptides from previous chemical glycoproteomics experiments**. (A) Chemical scar **2** is produced in an MOE-glycoproteomics experiment incorporating GalNAz as the sugar. The GalNAc-**2** oxonium ion is detected at 329.1455 m/z, along with two ions corresponding to the loss of water (311.1348, 293.1243). (B) Chemical scar **2Me** is produced in an MOE-glycoproteomics experiment incorporating GalNAz as the sugar. The GalNAc-**2Me** oxonium ion is detected at 343.1602 m/z. Another abundant ion is detected at 178.1972 m/z. Legend: y ions are indicated in purple. Oxonium ions are indicated in light green. Gal (yellow circle) = D-Galactose.


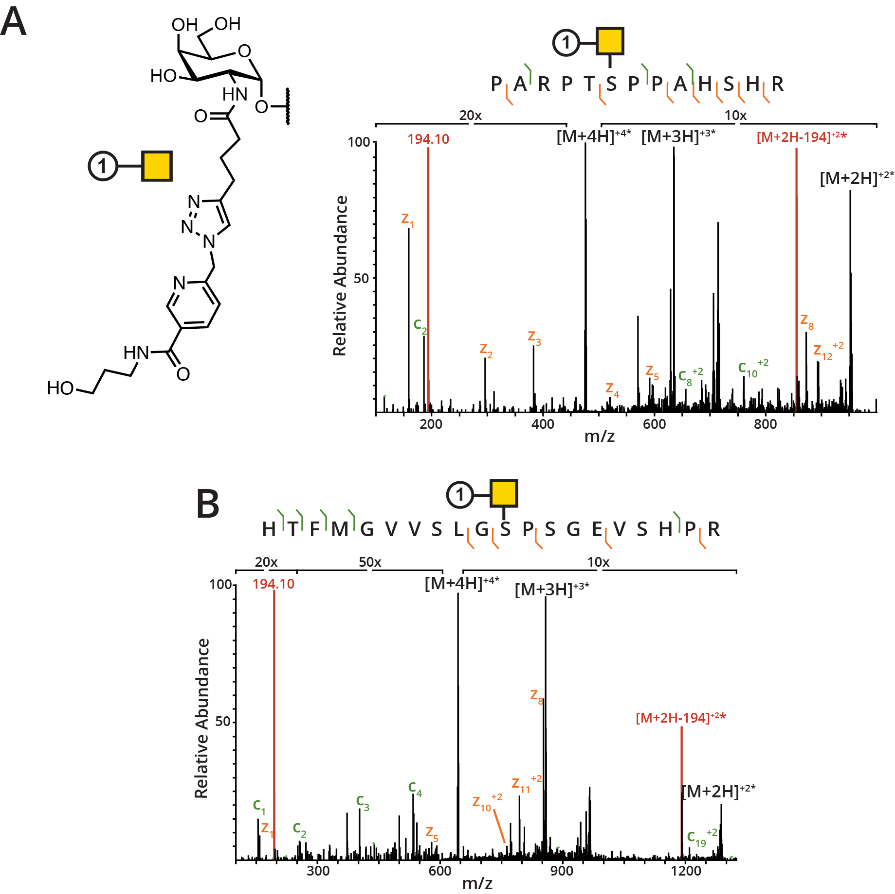


**Figure S2. Additional examples of GalNAc-1 ETD spectra.** GalNAc-**1** was produced in an MOE-glycoproteomics experiment incorporating GalN6yne as the sugar. The peptides were analyzed using an HCD-pd-ETD instrument method on an Orbitrap Fusion Lumos. (A) Structure of GalNAc-**1** (left) and a glycopeptide originating from collagen alpha-1(XVIII) chain (right). (B) a GalNAc-**1** modified peptide from alpha-2-HS-glycoprotein. In both of these spectra, note the presence of the 194.10 m/z ion as well as the small molecule fragmentation from the intact +2 peptide, noted in red. Z ions are denoted in orange, c ions are depicted in green.


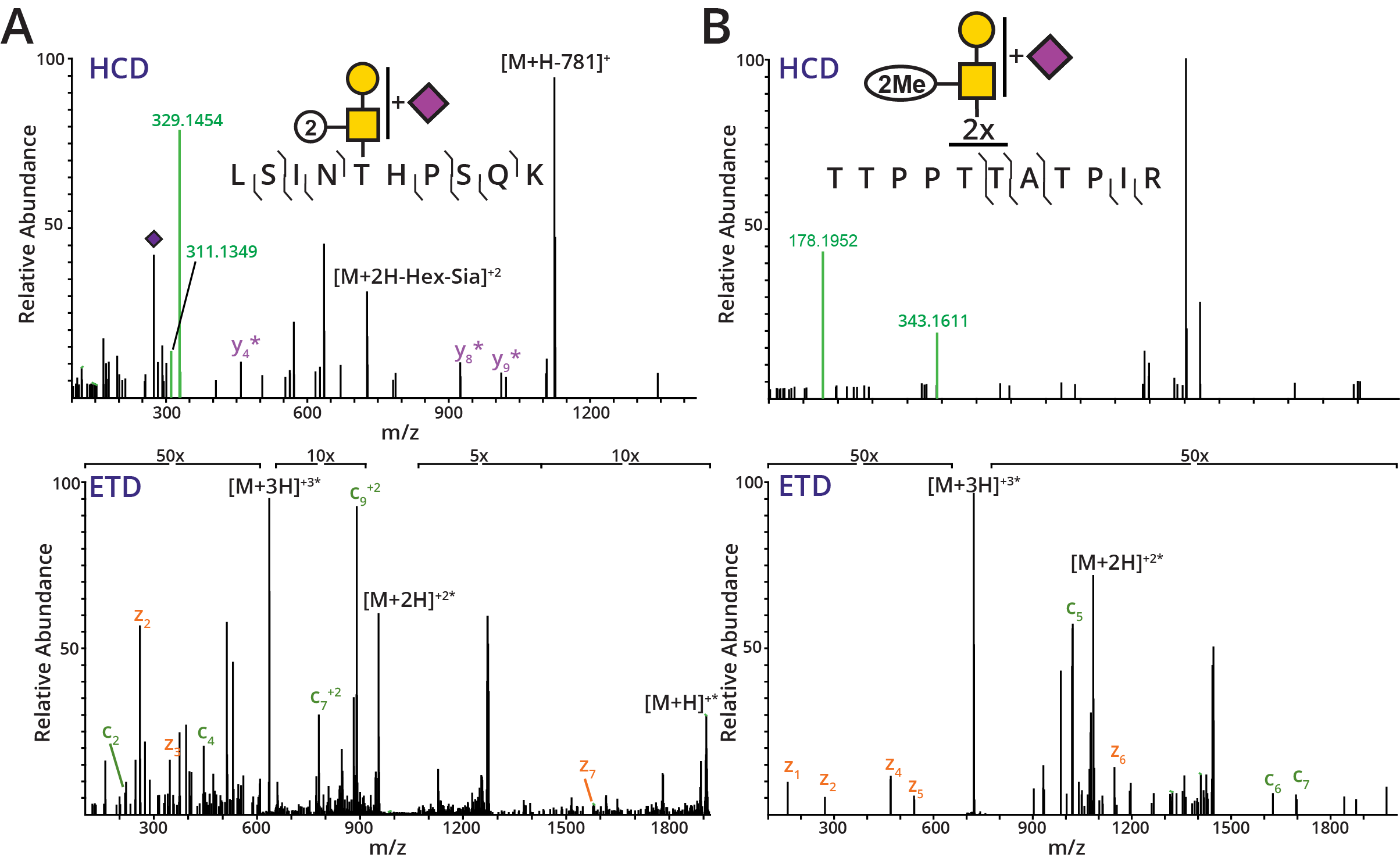


**Figure S3. Examples of GalNAc-2 and -2Me HCD/ETD spectra of elongated glycopeptides.** Chemical scars **2 and 2Me** were produced in an MOE-glycoproteomics experiment. The peptides were analyzed using an HCD-pd-ETD instrument method on an Orbitrap Fusion Lumos. HCD spectra (top) and ETD spectra (bottom) demonstrating that GalNAc analogs are extended by naturally occurring monosaccharides, such as galactose and sialic acid. (A) GalNAc-**2**-Hex-NeuAc glycopeptide from complement component C3. (B) Di-GalNAc-**2Me**-Hex-NeuAc peptide from fibronectin. Gal (yellow circle) = D-Galactose; Sia (purple diamond) = D-N-acetylneuraminic acid.


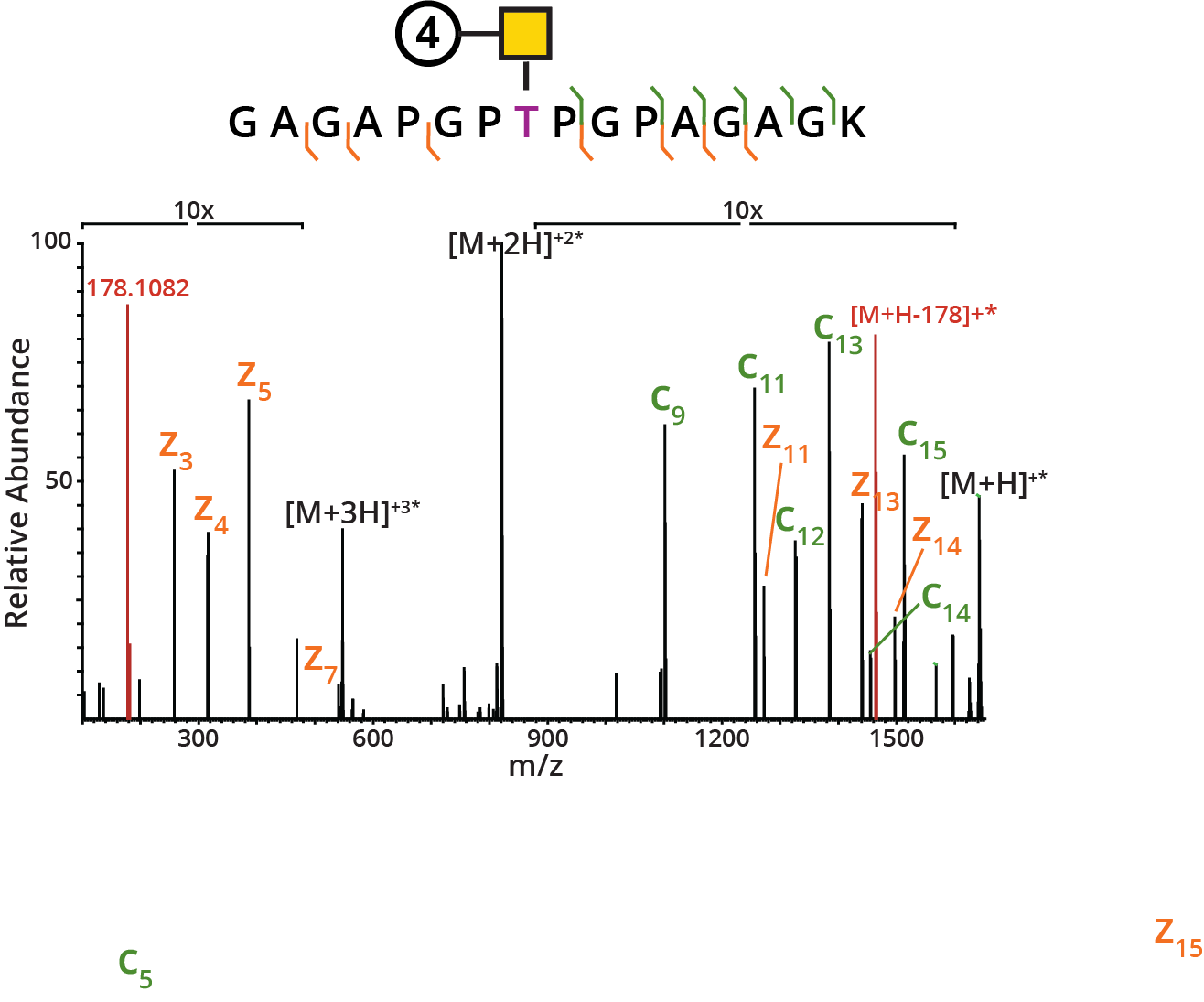


**Figure S4. ETD spectrum of GalNAc-4 acquired in the Orbitrap.** Synthetic glycopeptide was subjected to ETD and analyzed in the Orbitrap at 30K resolution at 400 FWHM. The site of modification is colored pink; z ions are denoted in orange, c ions in dark green, and ETD fingerprint ions in red. The accurate mass of the ETD fingerprint ion, as determined by this experiment, is 178.1082.

Scheme S1: Enzymatic synthesis of glycopeptides **B**, **C** and **D**.

Scheme S2: Synthesis of glycopeptides **E**, **F** and **G**.

Scheme S3: Synthesis of glycopeptides **H**, **I** and **J**.

**Methods**

The compound biotin-DADPS-picolyl-azide **SI-6** was purchased from Sussex Research (Ottawa, Canada). UDP-GalNAz was from Click Chemistry Tools, UDP-GalNAlk was synthesized previously.^1^

*Chemical synthesis of clickable ITag reagents* ***SI-1*** *and* ***SI-2***

Chemicals were commercial grade. Reactions were monitored by thin layer chromatography and detection was performed under UV light (254 nm) and by warming after staining with KMnO_4_ solution. Reverse phase chromatography was performed on Sep-Pak® Vac 20cc C8 columns. Reaction mixture residues were dried under vacuum using rotary evaporation and high vacuum oil pump. ^1^H NMR and ^13^C NMR spectra were measured in the solvent stated at 400 or 500 MHz. Chemical shifts are quoted in parts per million from residual solvent peak and coupling constants (J) given in Hertz. Multiplicities are abbreviated as: b (broad), s (singlet), d (doublet), t (triplet), q (quartet), m (multiplet) or combinations thereof. Mass analysis was performed by electrospray ionization (ESI-LRMS) on an UPLC-MS (Waters) or high-resolution MS (HRMS).

**1-(3-Butyn-1-yl)-3-methylimidazolium tetrafluoroborate (ITag-alkyne, SI-1)**

To a solution of 4-bromo-1-butyne (1.0 mL, 10.65 mmol), in anhydrous acetonitrile (20 mL) were added 1-methylimidazole (3.37 mL, 42.60 mmol) and KBF_4_ (5.36 g, 42.6 mmol). The mixture was stirred under inert atmosphere for 16 hours at 90 °C under reflux conditions. The reaction mixture was cooled to room temperature, filtered over a celite pad and the filtrate concentrated under reduced pressure. The residue was purified via silica gel column chromatography (0 to 15 % MeOH in DCM) furnishing ITag-alkyne **SI-1** (1.35 g, 5.2 mmol, 49%) as a white wax. ^1^H NMR (500 MHz, CD_3_OD) δ 9.08 (s, 1H), 7.73 (t, *J* = 1.8 Hz, 1H), 7.62 (t, *J* = 1.8 Hz, 1H), 4.40 (t, *J* = 6.4 Hz, 2H), 3.97 (s, 3H), 2.84 (td, *J* = 6.4, 2.6 Hz, 2H), 2.51 (t, *J* = 2.7 Hz, 1H); ^13^C NMR (126 MHz, CD_3_OD) δ 138.2, 125.0, 123.9, 79.9, 73.5, 49.3, 36.7, 21.1. HRMS (ESI) m/z: Calcd for C_8_H_11_N_2_^+^ (M)^+^ 135.0917, found 135.0916.

**1-(3-azidopropyl)-3-methylimidazolium 4-toluenesulfonate (ITag-azide, SI-2)**

To a solution of 3-azido-1-propanol (92 µL, 0.902 mmol) in anhydrous acetonitrile (4 mL) at 0 °C were added 4-toluenesulfonyl chloride (258 mg, 1.353 mmol) and 1-methyl-imidazole (720 µL, 9.02 mmol). The reaction was allowed to stir at 0°C for 3 hours until TLC showed complete consumption of the starting material (hexanes/EtOAc 4:1). The reaction was warmed to room temperature and KBF_4_ (1.135 g, 9.02 mmol) was added. The reaction was warmed to 80°C and stirred under inert atmosphere for 18 hours under reflux conditions. The reaction mixture was cooled to room temperature, filtered and the filtrate evaporated under reduced pressure. The residue was washed four times with diethyl ether (10 mL) under sonication. The solvent was evaporated and purified by reverse phase chromatography (water 0.1% formic acid, water 0.1% formic acid/acetonitrile 9:1). Fractions were checked by LC-MS, pooled and evaporated affording the product as a colorless oil (176 mg, 0.52 mmol, 58%). ^1^H NMR (400 MHz, CD_3_OD): 8.96 (s, 1H), 7.74-7.70 (m, 2H), 7.66 (t, *J* = 1.8 Hz, 1H), 7.59 (t, *J* = 1.8 Hz, 1H), 7.27-7.23 (m, 2H), 4.31, (t, *J* = 7.1 Hz, 2H), 3.94 (s, 3H), 3.45 (t, *J* = 6.4 Hz, 2H), 2.39 (s, 3H), 2.20-2.09 (m, 2H). ^13^C NMR (400MHz, CD_3_OD): 142.2, 140.3, 136.8, 128.4, 125.5, 123.7, 122.3, 47.7, 46.8, 35.1, 28.8, 19.88; HRMS (ESI) m/z: Calcd for C_7_H_12_N_5_^+^ (M)^+^ 166.1092, found 166.1086.

*Analysis of glycopeptides derived from GalN6yne, GalNAz and GalNAzMe from cellular MOE experiments*

Glycoproteomic samples and analyses were generated in Schumann et al., or as in Debets et al. and analyzed as described previously.^27,28^ Briefly, secretome samples of HepG2 cells with the capacity of introducing either GalN6yne, GalNAz or GalNAzMe into glycoproteins were treated with acid-cleavable, bioorthogonal biotin-containing molecules **SI-3** or **SI-6**, enriched on streptavidin or neutravidin matrices, subjected to on-bead proteolytic digestion and cleaved off the matrices with formic acid. Glycopeptide samples were analysed by mass spectrometry as described previously.^27,28^

*Expression and purification of GalNAc-T2*

Soluble GalNAc-T2 was expressed as described before,^2^ using a GalNAc-T2 expression plasmid derived from the pOPING vector, originally a gift from Ray Owens (Addgene plasmid # 26046; http://addgene.org/26046; RRID:Addgene_26046).^3^ Briefly, Expi293F^TM^ cells (Thermo Fisher) were diluted to 3x10^6^ cells/mL in 90 mL of Gibco® FreeStyle^TM^ Medium (Thermo Fisher) and transfected with a pOPING-GalNAc-T2 using the ExpiFectamine^TM^ 293 Transfection Kit (Thermo Fisher) according to the manufacturer’s instructions. Plasmid DNA (90 μg) was diluted in Opti-MEM Reduced Serum Medium (4.5 mL, Thermo Fisher) and mixed gently by inversion. ExpiFectamine^TM^ 293 Transfection Reagent (243 μL) was diluted in Opti-MEM Reduced Serum Medium (4.3 mL), mixed gently by inversion and incubated for 5 min at room temperature. The diluted transfection reagent and the diluted plasmid DNA were combined, mixed gently by inversion and incubated for 20 min at room temperature. The lipid-DNA complexes formed were added dropwise to the cells and incubated at 37 °C, 8% CO2 and 125 rpm for 24 h. The cells were then treated with ExpiFectamineTM 293 Transfection Enhancer 1 (450 μL) and ExpiFectamineTM 293 Transfection Enhancer 2 (4.5 mL) and incubated at 37°C, 8% CO2 and 125 rpm. After 4 days, the transfected cells were centrifuged (5 min, 500 rpm, 4°C) and the supernatant collected and filtered.

NiSO_4_ (1 mM, Sigma Aldrich) was added to the filtered supernatant and incubated for 15 min at 4 °C on a Tube Roller Shaker® (Stuart Equipment). Ni-NTA® Agarose beads (1 mL, Qiagen^TM^) were washed thrice with water and twice with Buffer A (50 mM Tris-HCl, 125 mM NaCl, 20 mM imidazole, pH 7.5), added to the filtered supernatant and incubated overnight at 4°C on a Tube Roller Shaker®. The beads were centrifuged (3 min, 2000 g, 4°C) and washed thrice with Buffer A (10 mL). The protein was eluted from the beads by addition of Buffer A (2 mL) with 20%, 40% and 60% (v/v) Buffer B (50 mM Tris-HCl, 125 mM NaCl, 500 mM imidazole, pH 7.5) in Buffer A. The eluted protein was dialysed using SnakeSkin^TM^ Dialysis Tubing (Thermo Fisher Scientific^TM^) against Dialysis Buffer (25 mM Tris-HCl, 125 mM NaCl, 20% glycerol, pH 7.5), quantified by Nanodrop Spectrophotometer (DS-11 Series, DeNOVIX), aliquoted and stored at -80°C.

*Chemoenzymatic synthesis of glycopeptides* ***B-D***

The glycosylation reaction was initiated by the addition of GalNAc-T2 (100 nM) to a reaction mixture containing 20.8 mM Tris-HCl pH 7.4, 50 mM NaCl, 10 mM MnCl_2_, 50 μM peptide **A**, 51 μM UDP-sugar (UDP-GalNAc, UDP-GalNAz or UDP-GalNAlk^1^) and 0.5 U Calf Intestinal Alkaline Phosphatase (CIAP, New England Biolabs, Ipswitch, USA). The glycosylation was conducted at 37°C for 42 h and quenched by heating at 95°C for 10 s. Reaction progress was monitored by taking aliquots, quenching by addition of an equal volume of ethanol and analyzing the supernatant by LC-MS. Turnover was > 75% for all glycopeptides as assessed by LC-MS.

*Synthesis of glycopeptides* ***E****,* ***G****,* ***I***

The enzymatic reactions containing glycopeptides **C** or **D** (20 μL) were treated with a CuAAC solution containing 600 μM BTTAA, 300 μM CuSO_4_, 5 mM sodium ascorbate, 5 mM aminoguanidinium chloride and 75 μM of reagents **SI-1** or **SI-3** (from glycopeptide **C**), or **SI-6** (from glycopeptide **D**) and incubated at room temperature. After 4 h, the same amount of the CuAAC solution was added t and incubated was continued overnight. The reaction mixtures were then treated with 2% (v/v) formic acid and incubated for 1 h at room temperature.

*Synthesis of glycopeptides* ***F****,* ***H****,* ***J***

The enzymatic reactions containing glycopeptides **C** or **D** (20 μL) were treated with a CuAAC solution containing 1200 μM BTTAA, 600 μM CuSO_4_, 10 mM sodium ascorbate and 100 μM of reagent **SI-4** (from glycopeptide **C**), or **SI-5** or **SI-7** (from glycopeptide **D**) and incubated at room temperature overnight. The reaction mixtures were then treated with 2% (v/v) formic acid and incubated for 1 h at room temperature.

**References**

(1) Choi, J.; Wagner, L. J. S.; Timmermans, S. B. P. E.; Malaker, S. A.; Schumann, B.; Gray, M. A.; Debets, M. F.; Takashima, M.; Gehring, J.; Bertozzi, C. R. Engineering Orthogonal Polypeptide GalNAc-Transferase and UDP-Sugar Pairs. *J. Am. Chem. Soc.* **2019**, *141* (34), 13442–13453. https://doi.org/10.1021/jacs.9b04695.

(2) Schumann, B.; Malaker, S. A.; Wisnovsky, S. P.; Debets, M. F.; Agbay, A. J.; Fernandez, D.; Wagner, L. J. S.; Lin, L.; Li, Z.; Choi, J.; Fox, D. M.; Peh, J.; Gray, M. A.; Pedram, K.; Kohler, J. J.; Mrksich, M.; Bertozzi, C. R. Bump-and-Hole Engineering Identifies Specific Substrates of Glycosyltransferases in Living Cells. *Mol. Cell* **2020**, *78* (5), 824-834.e15. https://doi.org/10.1016/j.molcel.2020.03.030.

(3) Kowarz, E.; Löscher, D.; Marschalek, R. Optimized Sleeping Beauty Transposons Rapidly Generate Stable Transgenic Cell Lines. *Biotechnol. J.* **2015**, *10* (4), 647–653. https://doi.org/10.1002/biot.201400821.

**NMR spectra**

^1^H NMR (500 MHz, CD_3_OD)

^13^C NMR (126 MHz, CD_3_OD)

^1^H NMR (400 MHz, CD_3_OD)


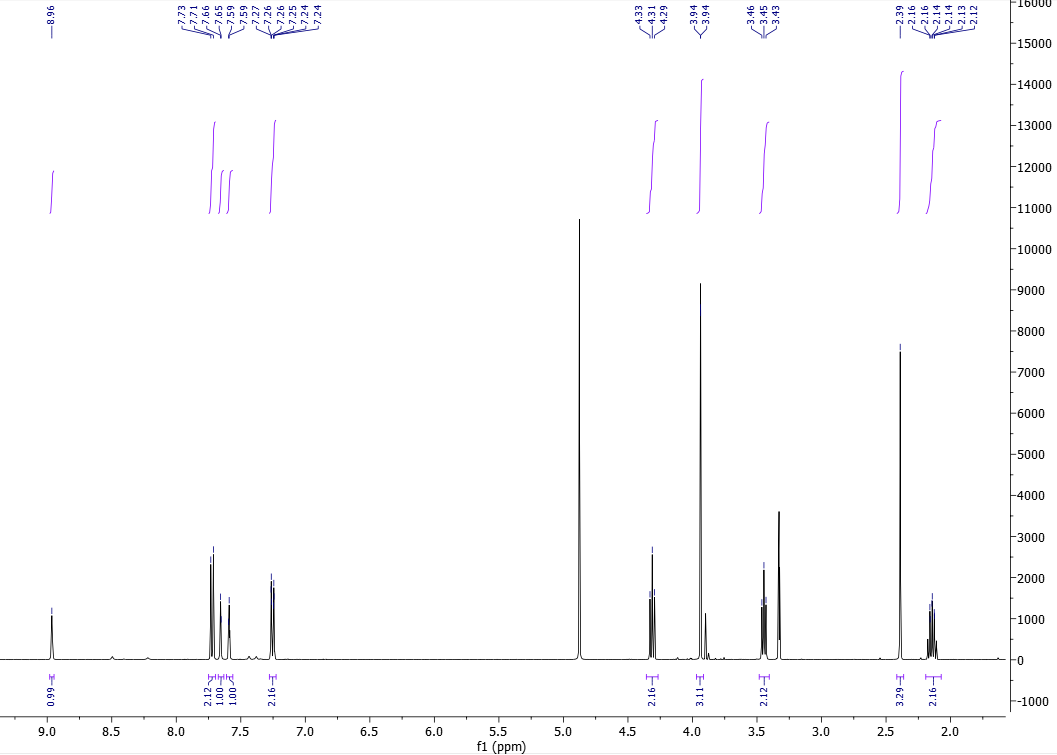


^13^C NMR (100 MHz, CD_3_OD)


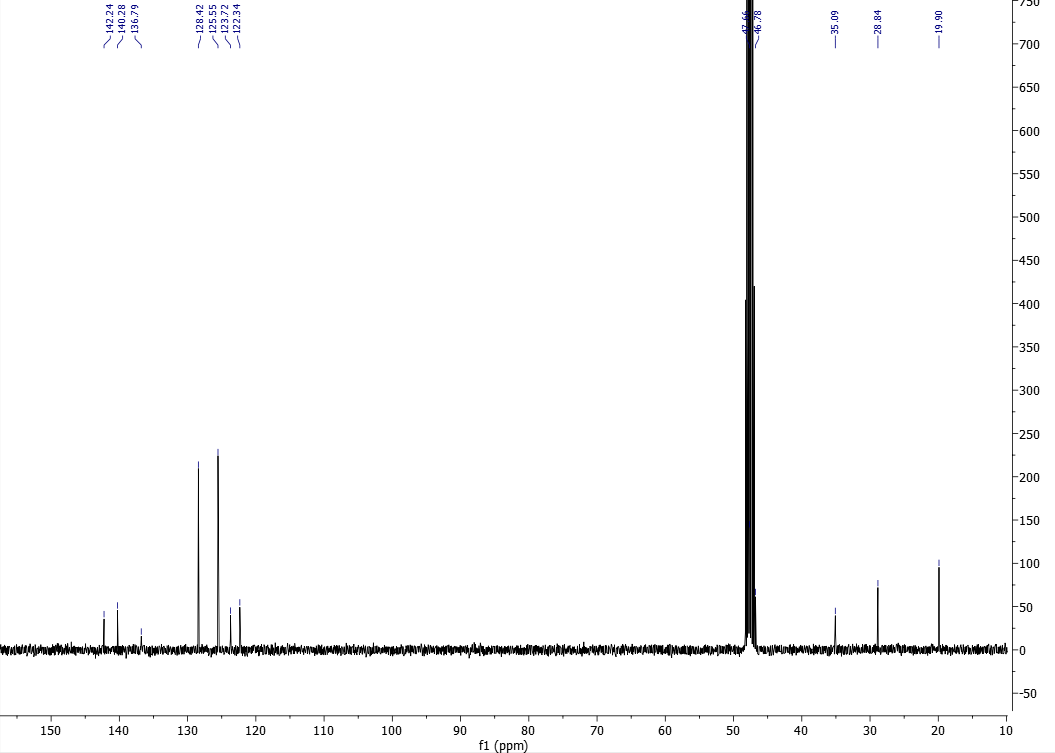

Supplement: Figure S1, Supporting Information [file EMS123017-supplement-Figure_S1__Supporting_Information.docx]
